# Early postoperative serum cystatin C predicts severe acute kidney injury following cardiac surgery: a post-hoc analysis of a randomized controlled trial

**DOI:** 10.1186/1749-8090-9-10

**Published:** 2014-01-08

**Authors:** Arndt-Holger Kiessling, Juliane Dietz, Christian Reyher, Ulrich A Stock, Andres Beiras-Fernandez, Anton Moritz

**Affiliations:** 1 Department of Thoracic and Cardiovascular Surgery, Johann Wolfgang Goethe University, Theodor Stern Kai 7, Frankfurt am Main 60590, Germany; 2 Department of Anaesthesiology and Intensive Care, Johann Wolfgang Goethe University, Frankfurtam Main, Germany

**Keywords:** Cystatin C, Cardiac surgery, Cardiopulmonary bypass, Acute kidney injury

## Abstract

**Objective:**

Acute kidney injury (AKI) after cardiac surgery procedures is associated with poor patient outcomes. Cystatin C as a marker for renal failure has been shown to be of prognostic value; however, a wide range of its predictive accuracy has been reported. The aim of the study was to evaluate whether the measurement of pre- and postoperative serum cystatin C improves the prediction of AKI.

**Methods:**

In a single-centre, prospective study of 70 patients (74 ± 9ys; range 47-85ys; 77% male), cystatin C was measured six times: (T1 = preoperative, T2 = start cardiopulmonary bypass (CPB), T3 = 20 min after CPB, T4 = end of operation; T5 = 24 h postoperatively; T6 = 7d postoperatively). Predictive property, in terms of the need for renal replacement therapy (RRT), was analysed by receiver operating characteristics (ROC) statistics and described by the area under the curve (AUC).

**Results:**

With respect to RRT (n = 8), serum cystatin C was significantly higher at the end of the operation (T4), 24 h postoperatively at T5 and at T6. The AUCs for preoperative T1 and intraoperative T2/3 cystatin C were <0.7 (95% CI, 0.47-0.85). The earliest significant predictive AUCs were found at the end of the operation (T4: p = 0.03 95% CI 0.58-0.88 AUC 0.73) and 24 h postoperatively (T5: p = 0.003 95% CI 0.74-0.96 AUC 0.85).

**Conclusions:**

Early postoperative serum cystatin C increase appears to be a moderate biomarker in the prediction of AKI, whereas a preoperative and intraoperative cystatin C increase has only a limited diagnostic and predictive value.

## Background

Particularly in the case of pre-existing renal disease and complex surgical procedures, acute kidney injury (AKI) is a frequently observed complication of heart surgery (5-10%) [1-3]. AKI is associated with increased mortality and ventilation time and longer stays in the ICU and hospital [2]. Criteria for diagnosing AKI are based on the relative or absolute serum creatinine fluctuations [4] and urine excretion. The disadvantage of these non-specific markers is their delayed appearance in particular. Serum creatinine is also affected by non-renal factors such as muscle mass, distribution volume and tubular secretion [4].

A series of new, alternative markers which are said to detect renal function as well as the appearance of acute insufficiency earlier and more accurately has been published. One of these new markers is cystatin C (CysC), a 13-kD cysteine protease inhibitor protein which is formed in all cells with a cell nucleus. In contrast to creatinine, it does not undergo tubular filtration but instead glomerular filtration and it is not subject to any significant protein binding. CysC has shown that it can accurately indicate even small changes in the glomerular filtration rate. However, various extrarenal factors can distort and affect cystatinC values. These include for example the thyroid function or the administration of glucocorticosteroids,. The mean reference serum cystatin C level is 0.84 mg/L in non-Hispanic adolescents and normal renal function [5]. It is not known how ultrafiltration during extracorporeal circulation affects serum CysC levels [6].

Studies were able to demonstrate that CysC is a good prognostic factor for the duration and severity of AKI following aortic valve replacement, coronary angiography and complex cardiac-surgery procedures [7-9]. The question of at what point postoperatively increases in CysC are predictive in nature is unclear. From this we formulated the main question of the study: from when and with which RIFLE definition [10] can CysC levels predict AKI in patients with cardiopulmonary bypass (CPB) (with and without intraoperative ultrafiltration).

## Methods

### Patients

This study is a post-hoc analysis of a study that has already been published which was primarily designed to detect differences in the preoperative coating of the CPB (NCT01247051). We recruited 70 patients with elective on-pump cardiac-surgery procedures at the Johann Wolfgang Goethe University Hospital (Frankfurt am Main, Germany) in the period from the first quarter of 2011 to the third quarter of 2011. A written informed consent form was obtained from all patients. The study was appoved by the local ethic committee (No:143/10). Inclusion criteria were patients with normal renal function,age of >75 years and/or a reduced left ventricular function of <40%. Furthermore, informed consent and elective, planned surgery using the heart-lung machine was needed. Patients with combined interventions on the aorta and/or carotid arteries and/or multiple valve operations were excluded. Patients were similarly not included into the study if the following preoperative exclusion criteria were present: serum creatinine >1.8 mg/dl, raised GOT/GPT values double the norm, haemoglobin (Hb) <11 mg/dl, myocardial infarction within the last seven days before surgery and affiliation to the religious community of the Jehovah’s Witnesses [11].

### Data and sample collection

The preoperative patient data were collected 24 hours before the surgery. In addition to routine clinical care parameters, they also covered study-specific parameters which can affect the cystatin value. These included the GFR, administration of cortisones, thyroid function values (TSH), and C–reactive protein (C-rP). Intraoperatively, CPB parameters (cardiac index, CPB time, ACC time, urine excretion, type of ultrafiltration) were added to the data. Furosemide and exogenous steroid administration was recorded until the second postoperative day. The postoperative volume status was calculated using patient weight and fluid balance.

### Laboratory testing

Six measurement time points (T1 preoperative, T2-4 intraoperative, T5-6 postoperative) were defined. The blood samples were stored on ice and centrifuged at 4°C at 4000 rpm for 15 minutes. The serum samples were then stored at −20°C. The analysis was performed in the central laboratory of the Centre for Internal Medicine and the study laboratory of the Department of Thoracic and Cardiovascular Surgery of Johann Wolfgang Goethe University hospitals in Frankfurt am Main (recognition according to DIN EN ISO 15189; DIN EN ISO 9001:2008 accreditations of medical laboratories). Serum cystatin C was determined by means of an immunoassay (Bühlmann, Germany). The lower limit of detection was 0.05 mg/L. The clinical parameters are based on the evaluations of the German federal cross-sector quality assurance association (SQG, Aqua Institut Göttingen, Germany) and were taken from existing postoperative databases.

### Endpoint definition

The primary endpoint was the new occurrence of acute kidney injury within 48 h after the end of the surgery. We chose a brief observation period, since prior studies have demonstrated that AKI occurs within the first 24-48 h after use of extracorporeal circulation [12]. In the primary analysis, we defined AKI as the use of renal replacement therapy (RRT-AKIN3). In a secondary analysis, AKI was defined as an increase of 50% or 0.3 mg/dL over the baseline creatinine value (AKIN1). This interpretation corresponds to the Acute Kidney Injury Network criteria [4]. An additional AKI criterion of the Acute Kidney Injury Network is oliguria (urine excretion <0.5 mL/kg/h for 6 hours) [4]. This criterion is difficult to describe in the postoperative, cardiothoracic care. Due to intravenous fluid administration and diuretic application, the diagnosis of an AKI by oliguria could be masked by the individual ICU therapies. The urine output was nevertheless recorded at all measurement time points [10].

### Statistical analysis

Continuous variables with normal distribution were evaluated using the unpaired *t* test and variables with non-normal distribution were evaluated using the Wilcoxon test. Categorical variables were measured with the χ2 and Fisher’s exact test. CysC was compared at each measurement time point with the unpaired *t* test. Serial CysC measurement values were compared in patients with and without AKI with a linear mixed correlation. We calculated receiver operating characteristics (ROC) in order to describe the correlation of CysC. The area under the curve (AUC) with an associated 95% confidence interval (CI) was used as a measurement for the discriminating capacity of CysC to predict AKI. An ROC AUC value of 0.60-0.69 demonstrates a poor predictive value, 0.70-0.79 a moderate predictive value, 0.80-0.89 a good predictive value and 0.90-0.99 an excellent predictive value. Logistic regression was calculated to evaluate whether increasing CysC values are implicated, independently of the development of an AKI. Preoperatively, we defined variables which are implicated as confounders of AKI [1]. The final model was calculated in incremental methods of selection whereby the inclusion of a variable with P < 0.10 in the model was permissible. Since CysC can be increased in patients with chronic renal disease (GFR <60 mL (min/1.73 m^2^), these data were not included in the analysis. All analyses were calculated using the IBM SPSS version 20.0 (New York, USA). A two-sided P value of <0.05 was considered to be statistically significant.

## Results

### Clinical outcome

All blood draws were able to be performed on 70 patients. Three other patients were not included in the analysis. This was due to a lack of preoperative blood values. Twenty-one patients developed AKI according to creatinine criteria (AKIN 1); 8 patients had to be treated with renal replacement therapy. Preoperative, baseline and postoperative characteristics are shown in Table 1. CPB time and aortic clamping time were significantly longer in patients who developed AKI. Patients with AKI were older (p = 0.003) and demonstrated a trend towards higher preoperative creatinine values (p = 0.13). Patients with AKI have a poorer clinical outcome, including length of stay in the ICU and hospital (Table 1). The indications for and timing of a dialysis procedure were not standardised in the protocol and were individual medical decisions. The main reason for an acute dialysis therapy was an anuresis in the first postoperative hours (n = 4) after the cardiac procedure. Renal replacement therapy was initiated after mean of 2.1 ± 1.9 days (range 0-4days) and terminated after 22.5 ± 21 days (range 2-61days). Non of patients went into a chronic program. The other dialysis indications were potassium overload (n = 1) and respiratory failure due to lung congestions (n = 3).

**Table 1:** Comparison of patients with Acute kidney injury (renal replacement therapy) and patients without any renal complications

	**No AKI**	**AKI (renal replacement)**	**p-value**
Baseline			
Male% (n)	64(40)	75(6)	0.79
Age ys	72.6 ± 10.1	80 ± 3.7ys	0.003
Weight kg	83 ± 15	87 ± 14	0.51
Baseline Crea mg/dl	1.09 ± 0.3	1.6 ± 0.84	0.13
Baseline GFR stage	2.2 ± 0,6	2.25 ± 0,6	0.8
Elective% (n)	100(62)	100(8)	
ASA class ≥II% (n)	34(21)	62(5)	0.13
Diabetes% (n)	29(18)	37(3)	0.45
LV function reduced% (n)	53(33)	37(3)	0.47
Surgical data			
Bypass time min	116 ± 39	155 ± 38	0.01
Cross-clamp time min	72 ± 32	102 ± 36	0.02
Urine output on bypass ml	360 ± 290	315 ± 310	0.32
Post-filtration CPB% (n)	8(5)	37(3)	
CABG% (n)	62(39)	25(2)	0.05
Postoperative data			
Fluid balance 24 h ml	1103 ± 1026	1403 ± 860	0.001
Furosemide total dose 48 h (mg)	90 ± 60	98 ± 65	0.4
Secondary outcome			
Length ICU stay d	1,58 ± 1.89	23.25 ± 20.88	0.000
Length hospital stay d	11 ± 5.9	30.6 ± 19.1	0.000
Mortality 30d% (n)	6.4(4)	25(2)	0.1

Significant differences were found in CPB times, length of hospital and ICU stay and age.

### Cystatin C performance

**Figure 1:**
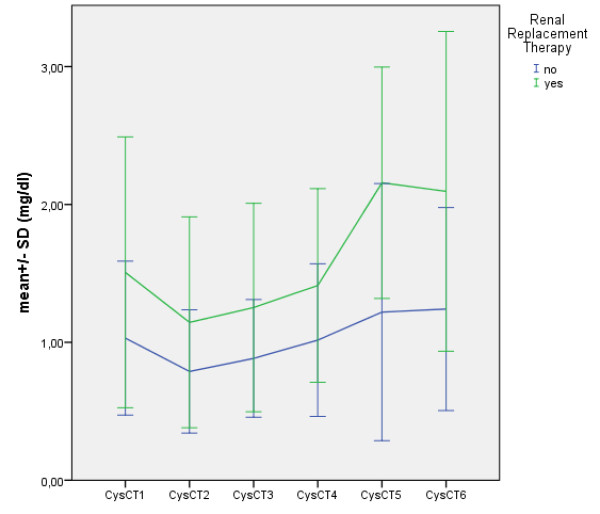
Shows the longitudinal differences between time (t1-t6) and the measured CysC values in patients with and without postoperative renal replacement therapy.

In a linear mixed model in a paired comparison, CysC values significantly differ in patients with and without AKI starting at time point T4 (end of operation). The difference becomes increasingly more significant with the time from the operation (Tables 2 and 3). Figure 1 shows the longitudinal differences between time (t1-t6) and the measured CysC values. Based on the baseline values, there were significant differences for CysC at times T4/5/6 (Table 3). Patients with AKI have a higher CysC level over all measurement time points; however, the earliest predictor of AKI was seen at time T4 (end of operation). In the ROC analysis, CysC peaked on postoperative day 4 with 0.733 (95% confidence interval, 0.586-0.88). All earlier times were poorer predictors of CysC and AKI (0.7). All measurement times after T4 had a higher predictive value. (T5 = 0.84, 95% CI, 0.739-0.960/T6 0.899, 95% CI, 0.779-1; all p-values <0.05). On the other hand, if patients with AKI are defined not only with the hard criterion of renal replacement therapy, but if patients with a 50% increase in creatinine are added (AKIN 1), then the size of the group increases from 8 to 21. With the increase in the number of patients, however, the predictive value of CysC also decreases. At no time is there a moderate to good predictive value (ROC >0.8) for the connection between CysC and the expanded definition of AKI (Table 4). Highly significant correlations (p < 0,001) with serum creatinine (Scr) and blood urea nitrogen (BUN) were found at any time points Table 5. These comparisons suggest that CysC is not superior to the routine markers (Scr/BUN).

**Table 2:** CystatinC kinetics in patients with AKIN1

**Collection time**	**AKIN 1**	**Mean**	**Std**	**p-value**
T1 preoperative	No	1,02	,27	0,29
	Yes	1,11	,36	
T2 start CBP	No	,79	,18	0,16
	Yes	,88	,31	
T3 20 minutes after CBP start	No	,88	,20	0,28
	Yes	,95	,28	
T4 OP-end	No	,99	,26	01
	Yes	1,12	,28	
T5 24 h postoperative (POD1)	o	1,20	,48	0,04
	Yes	1,63	,60	
T6 discharge (max. day 6)	No	1,25	,40	0,02
	Yes	1,62	,56	

Late significant differences were found on POD 1 and discharge (POD 6).

**Table 3:** CystatinC kinetics in patients with renal replacement therapy

**Collection time**	**AKI (renal replacement)**	**Mean**	**Std**	**p-value**
T1 preoperative	No	1,0	,27	0,06
	Yes	1,24	,43	
T2 start CBP	No	,80	,21	0,07
	Yes	,96	,31	
T3 20 minutes after CBP start	No	,89	,21	0,06
	Yes	1,05	,32	
T4 OP-end	No	1,00	,26	0,03
	Yes	1,22	,30	
T5 24 h postoperative	No	1,26	,52	0,002
	Yes	1,93	,47	
T6 discharge(max. day 6)	No	1,28	,40	0,001
	Yes	2,09	,57	

Early significant differences were found at the end of operation (p = 0.03).

**Table 4:** Moderate to good prognostic AUC values for CystatinC were found in patients with renal replacement therapy at T4 to T6

**CystatinC (N = 70) Time**	**AKI (AKIN 1)**	**AKI (renal replacement therapy**
T1 preoperative	0,553 (CI:0,4-0,7)	0,659 (CI:0,47-0,84)
T2 start CBP	0,565 (CI:0,41-0,71)	0,671 (CI:0,5-0,84)
T3 20 minutes after CBP start	0,528 (CI: 0,37-0,67)	0,650 (CI:0,45-0,84)
T4 OP-end	0,63 (CI:0,49-0,77)	0,733 (CI:0,58-0,88)
T5 24 h postoperative	0,72 (CI:0,58-0,86)	0,849 (CI:0.73-0,96)
T6 discharge (max. day 6)	0,7 (CI:0,53-0,87)	0,899 (CI:0,77-1,0)

**Table 5:** Good prognostic AUC values for creatinine were found in patients with renal replacement therapy at any timepoint

**Creatinine (N = 70) Collection time**	**AKI (renal replacement therapy)**
TX1 postoperative day 1	0,876 (CI:0,72-1,00)
TX2 postoperative day 2	0,904 (CI:0,797-1,00)
TX3 postoperative day 3	0,904 (CI:0,784-1,00)
TX4 postoperative day 4	0,928 (CI:0,816-1,00)

### CysC and influencing factors

Ultrafiltration, priming of the CPB and fluid balance do not affect the kinetics of CysC. In all patients, the preoperative baseline value falls by a median 20% after the start of the CPB and increases back up to the baseline level by the end of the operation, independently of the perfusion methods used. Then, in the following 48 h, the values increase (median 23%) above the preoperative baseline value.

Eleven patients received intra- and early postoperative corticosteroids. There was no connection between the steroid dose and CysC at T5 or later T6. No increase in CysC was detected in these patients. In nearly all patients, preoperative TSH values demonstrated a normal thyroid metabolic status. Analyses outside of the 95% confidence intervals for extreme values could not provide any evidence that high or low TSH values influence CysC kinetics.

## Discussion

Following cardiac-surgery procedures, serially measured serum CysC concentrations are positively associated with the onset of postoperative renal failure. CysC values measured immediately at the end of the operation provide an early indication of the possible onset of AKI. However, the predictive value depends on the AKI definition, according to whether renal replacement therapy or the increase in creatinine (AKIN 1) is used for this purpose. This may also explain the weakness of CysC, namely that it is not possible to predict before surgery whether the patient will develop AKI postoperatively.

Our results are comparable with those of other studies which confirm the reliability of CysC as a marker of renal function [13,14], particularly when an CPB is used [15-17]. Our data support the expanded indication of CysC as a marker of glomerular filtration as well as of chronic and acute renal insufficiency. CysC is an attractive marker for assessing GFR [17]. CysC is synthesized by all nucleated cells and, in contrast to creatinine, which is produced by muscle cells, CysC is not affected by the interindividual differences and intraindividual changes in the muscle mass. In addition, CysC is filtered at glomerular level and metabolised without undergoing tubular secretion or reabsorption. It is noteworthy that CysC concentrations differ in patients with and without AKI at all time points. The decrease during CPB can be explained by haemodilution [18] and for the immediate postoperative increase there is a correlation between CysC and C-reactive protein as a direct marker of CPB-induced inflammation [19].

Current studies remain unclear with regard to the benefit of CysC, however. Our finding regarding a limited benefit of CysC as an early marker of AKI is identical to the observations of Koyner et al. [20]. The CysC values of 70 patients with CPB were determined upon arrival in the ICU. With an AUC for the development of AKI (defined as a serum creatinine increase of 50% as compared to the baseline value) of 0.62 (95% CI, 0.48 to 0.75), there is a slight CysC-AKI association. This can be explained by the work of Rosner et al. and Moran [2,21], who see CPB-associated AKI as being mediated by damage to the renal tubules, whereas CysC is a marker of glomerular filtration. As a result of tubular damage, upstream glomerular filtration can be impacted due to congestion and can explain an increase in CysC.

However, other studies support the utility of CysC as an early predictor of AKI. In a cohort of critically ill patients, serum cystatin C demonstrated the occurrence of a creatinine-based AKI 1 to 2 days earlier than an established marker [22]. In a recently published study of 100 patients with CPB, the predictive capacity of plasma CysC upon arrival at the ICU was excellent (AUC 0.83, 95% CI, 0.68 to 0.98) [23]. The reasons for the discrepancy are not entirely clear. On the one hand, the perfusion techniques may differ with regard to haemodilution and post-filtration. On the other hand, the definition of AKI remains very variable and, finally, the CysC measurement times are different. A meta-analysis by Zhang et al. [23] was able to identify 15 studies which investigated the predictive value of CysC. A high OR of 23.5/95% CI, 14.2-38.9 = for serum CysC was found across all different study endpoints. Five studies were performed in a cardiac-surgery population. The pooled AUC was 0.96 (95%0.95.0.97), which corresponds to a very good correlation with AKI. In this review, Zhang concluded that serum CysC is an outstanding predictor of the onset of AKI, irrespective of the population investigated (sepsis, cardiac surgery). The superiority to serum creatinine is the non-dependence on age, sex, muscle mass and earlier onset. In a multicentre, prospective study of the Tribe-AKI (Translational Research Investigating Biomarker endpoints for Acute Kidney Injury) consortium [24] published in July 2012, it was found, in contrast to Zhang [25], that in the case of creatinine-defined AKI (cardiac surgery), sensitivity and specificity were lower for CysC than for serum creatinine. However, the sampling times differ from our results. Spahillari et al. [24] did not collect blood samples until 24 h after the operation but concluded that there were no advantages of CysC over CREA. The limitations of the study are, firstly, patient selection. Due to the post-hoc sub-analysis, no urgent and complex procedures (e.g. triple valves, aortic surgery, redo’s) were included in the evaluation. On the other hand, blood sampling times in a narrower postoperative interval would have been of use. AKI was only defined by creatinine increase and not by the second criteria of a decreasing urine output. Patients who develop AKI have higher rates of mortality and resource utilization, with the worst values seen in dialyzed patients. Emerging evidence suggests that even small changes in creatinine after cardiac surgery are associated with significant effects on mortality. Whether AKI directly causes adverse outcomes is not entirely clear; however, an increase in infection and new-onset sepsis, congestive heart failure, and fluid overload may be contributory [1]. Traditional biomarkers of AKI (creatinine and urea) increase slowly in response to renal injury, are insensitive to mild degrees of AKI, and are influenced by nonrenal factors. There is considerable interest in novel biomarkers of AKI such as cystatine c that increase rapidly after renal injury, detect mild degrees of AKI, and are less subject to nonrenal factors. It has been postulated that the early diagnosis of cardiac surgery-associated AKI using novel biomarkers will result in improved outcomes. However, there is little evidence that interventions started early in the course of evolving AKI enhance renal recovery. Until effective therapies are developed that significantly improve the outcome from AKI, there is little benefit from early diagnosis using novel biomarkers [25,26].

## Conclusion

In summary, we were able to calculate a subanalysis of the connection between CysC and AKI in our prospective study. The early predictive value can only be evaluated when AKI is defined as renal replacement therapy. By contrast, in the case of an AKIN 1 stage (increase in creatinine >50% baseline value), no good statistical connections can be detected. Cystatin can be determined as an additional parameter in cardiac-surgery procedures; however, the cost-effective and simple determination of the serum creatinine value continues to be the routine recommendation. A prospective, randomised study with prophylactic dialysis as an indication in one study arm may potentially provide a new starting point for the validity of CysC in clinical use.

## Competing interests

The authors declare that there is no conflict of interests.

## Authors’ contributions

Kiessling: Conceived and designed the study, analysis and interpretation of data and coordi-nation and wrote the manuscript. Dietz: Participated in patient- and sample recruitment and data completition. Reyher has been involved in the intraoperative and ICU therapy interpretation and revising it critically for important intellectual content. Stock, Beiras-Fernandez and Moritz, conceived the study, coordination and revising it critically for important intellectual content and reviewed the manuscript. All authors read and approved the final manuscript.
